# Supplementary material for: A Novel Vaccine Strategy to Prevent Cytauxzoonosis in Domestic Cats
Source: Vaccines (Basel). 2023 Mar 2;11(3):573. doi: 10.3390/vaccines11030573 (PMC10058880; doi:10.3390/vaccines11030573)
Supplement: Supplementary file 1 [file vaccines-11-00573-s001.zip › vaccines-2230484-supplementary.pdf]

Supplementary Figures

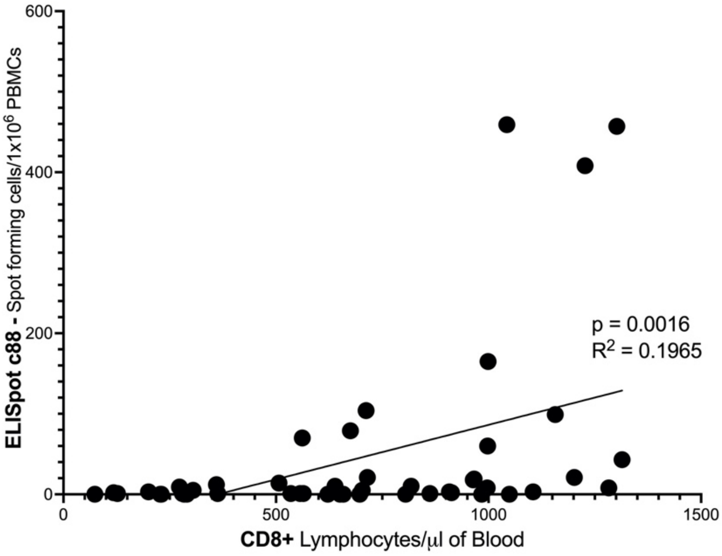

Figure S1. A significant correlation was observed between CD8+ cells/ul of blood and the cell-mediated immune response to vaccine epitope c88.

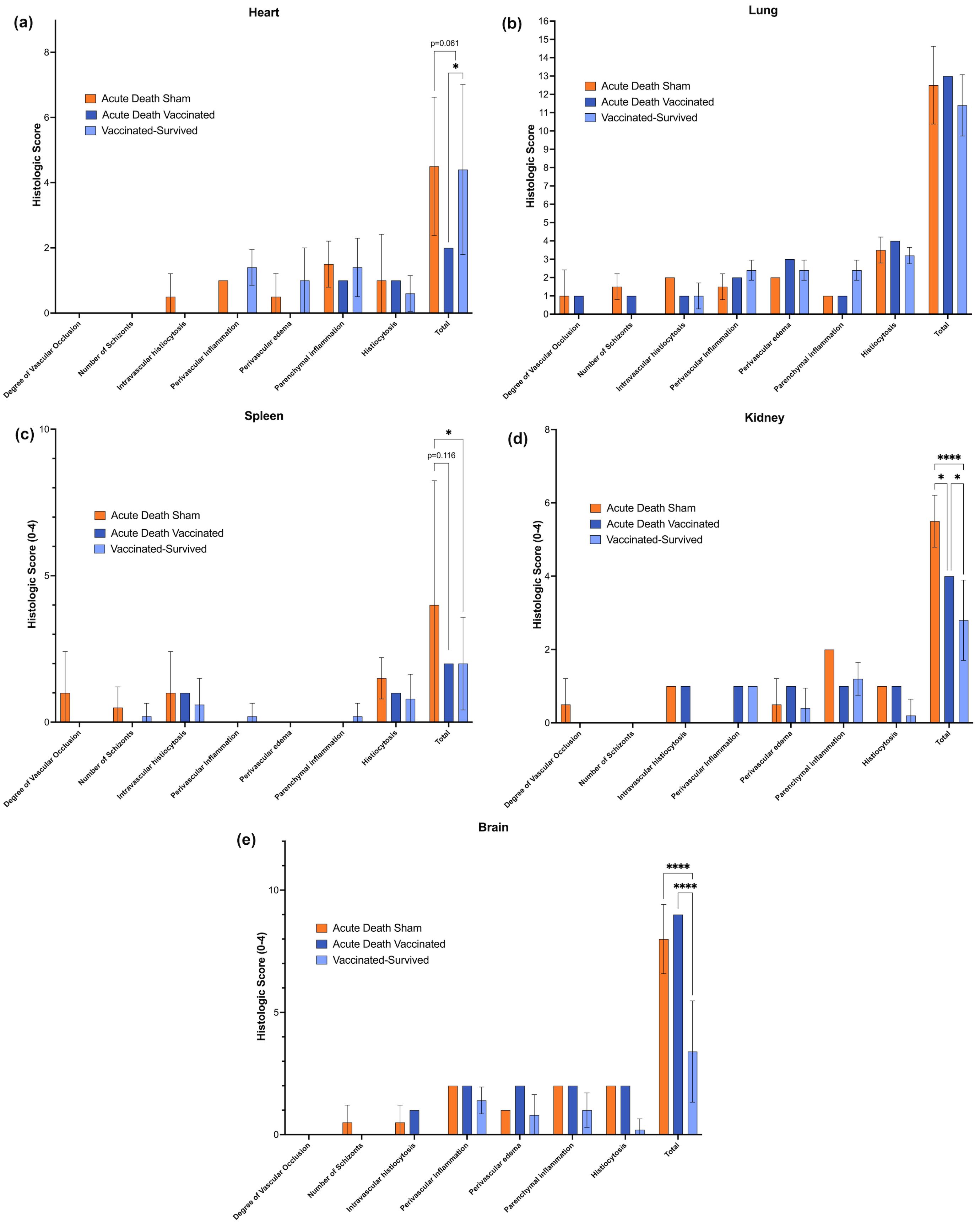

Figure S2. Average histological scores of the cats following *C. felis* infection in: (a) heart; (b) lung; (c) spleen; (d) kidney; (e) brain. ****=p<0.0001; *=p<0.05.
